# Assessment of a Single Quadrupole Mass Spectrometer Combined with an Atmospheric Solids Analysis Probe for the On-Site Identification of Amnesty Bin Drugs

**DOI:** 10.1021/jasms.4c00064

**Published:** 2024-06-05

**Authors:** Anca Frinculescu, Benjamin Mercer, Trevor Shine, John Ramsey, Lewis Couchman, David Douce, Nunzianda Frascione, Vincenzo Abbate

**Affiliations:** † Department of Analytical, Environmental and Forensic Sciences, King’s College London, 150 Stamford Street, London SE1 9NH, United Kingdom; ‡ TICTAC Communications Limited, Room 1.159 Jenner Wing, St. George’s University of London, Cranmer Terrace, London SW17 0RE, United Kingdom; § Clinical Pharmacology, William Harvey Research Institute, Queen Mary University of London, London EC1M 6BQ, United Kingdom; ∥ Analytical Services International, St. George’s University of London, Cranmer Terrace, London SW17 0RE, United Kingdom; ⊥ Waters Corporation, Stamford Avenue, Wilmslow SK9 4AX, United Kingdom

## Abstract

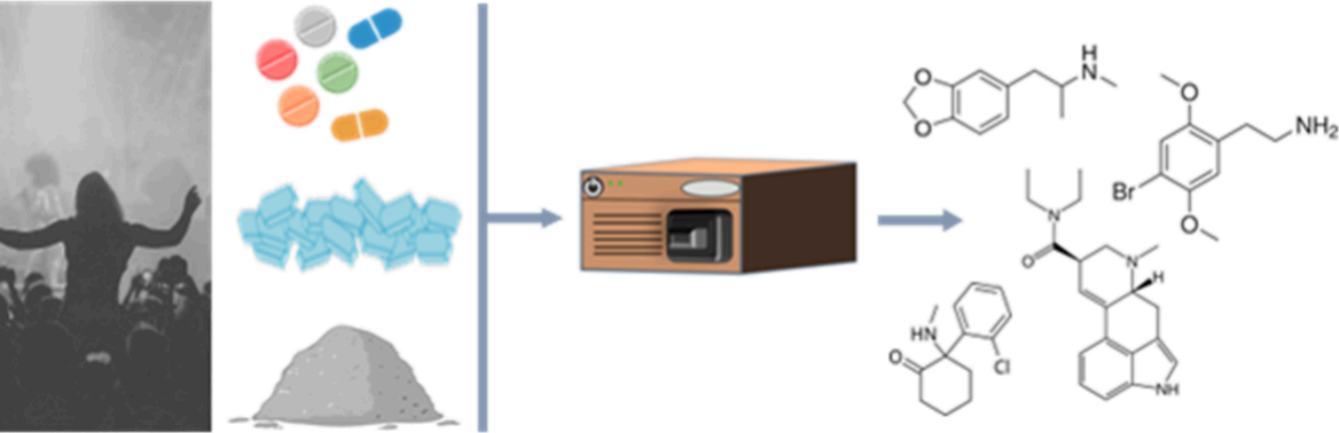

The surging number of people who abuse drugs has a great impact on healthcare and law enforcement systems. Amnesty bin drug analysis helps monitor the “street drug market” and tailor the harm reduction advice. Therefore, rapid and accurate drug analysis methods are crucial for on-site work. An analytical method for the rapid identification of five commonly detected drugs ((3,4-methylenedioxymethamphetamine (MDMA), cocaine, ketamine, 4-bromo-2,5-dimethoxyphenethylamine, and chloromethcathinone)) at various summer festivals in the U.K. was developed and validated employing a single quadrupole mass spectrometer combined with an atmospheric pressure solids analysis probe (ASAP-MS). The results were confirmed on a benchtop gas chromatography–mass spectrometry instrument and included all samples that challenged the conventional spectroscopic techniques routinely employed on-site. Although the selectivity/specificity step of the validation assessment of the MS system proved a challenge, it still produced 93% (*N* = 279) and 92.5% (*N* = 87) correct results when tested on- and off-site, respectively. A few “partly correct” results showed some discrepancies between the results, with the MS-only unit missing some low intensity active ingredients (*N*-ethylpentylone, MDMA) and cutting agents (caffeine, paracetamol, and benzocaine) or detecting some when not present. The incorrect results were mainly based on library coverage. The study proved that the ASAP-MS instrument can successfully complement the spectroscopic techniques used for qualitative drug analysis on- and off-site. Although the validation testing highlighted some areas for improvement concerning selectivity/specificity for structurally similar compounds, this method has the potential to be used in trend monitoring and harm reduction.

## 1 Introduction

Recreational use of controlled substances has been a perpetual concern to authorities and a significant burden to healthcare professionals and law enforcement alike, causing the loss of valuable resources.^1^ According to the Crime Survey for England and Wales (CSEW, 2022), 1 in 11 adults aged 16–59 and approximately 1 in 5 adults aged 16–24 admitted using drugs in the previous year.^2^ These already alarmingly high numbers are exacerbated in nightlife settings, which are known to have a strong association with recreational drug use.^3^ Despite nightclubs, concert venues, and music festivals operating a zero-tolerance policy on drug use, the prevalence of controlled substances at these events remains high.^4−6^ Therefore, monitoring drug trends in these settings (i.e., music festivals and nightclubs) could shed some light on the overall “street drug” market. Moreover, on-site drug analysis at festivals is a potential tool for preventing and reducing harm, allowing festival organizers to warn attendees about any particularly dangerous substances that may be in circulation and to inform medical and welfare staff.^7,8^ The principal means of collecting this type of data is through the analysis of the contents of amnesty bins. These are secure, locked boxes typically placed at the entrances to venues, to encourage attendees to safely dispose of controlled items without repercussions.^9^ The analysis of their contents does not only help with trend monitoring and harm reduction, but also can indicate new drug use habits and detect mis-sold drugs.^10,11^

Two important requirements for on-site drug analysis are portability and speed. Therefore, the techniques of choice for most drug-checking organizations are Fourier transform infrared (FTIR) and Raman spectroscopy.^12,13^ Although these are fast, nondestructive, and portable, they are limited to single-component samples, with mixture analysis and low-concentration samples remaining a challenge.^14^ Consequently, the “gold-standard” analytical method for drug analysis is gas or liquid chromatography coupled with mass spectrometry (GC- or LC-MS). The combination of a chromatographic separation technique with mass-selective detection allows the identification of multiple components present in samples. Furthermore, these techniques are sufficiently sensitive, reproducible, and can be both qualitative and quantitative.^15,16^ Although GC-MS instruments are not typically portable, some systems have been developed and purpose-built to be used in the field, including for analysis of illicit drugs.^17−20^ However, when compared to spectroscopic methods, the chromatographic step means longer analysis times (7–15 min) hence low sample throughput, and the initial price and service costs make them potentially less viable for on-site drug analysis.^21^ That said, the advances in portable MS (including ambient ionization MS where no chromatographic step is used) have shown potential for identifying illicit compounds in samples without requiring significant knowledge or training in MS.^17,22−27^

This study aimed to develop and validate an analytical method for the rapid identification of five of the most common drugs seen at several summer British festivals [3,4-methylenedioxymethamphetamine (MDMA), cocaine, ketamine, 4-bromo-2,5-dimethoxyphenethylamine (2C-B), and chloromethcathinone (CMC)] using a single quadrupole MS combined with an atmospheric pressure solids analysis probe (ASAP) (Radian ASAP, Waters, Wilmslow, U.K.). This was evaluated by comparison with a laboratory-based GC-MS instrument. The ability of the Radian ASAP to identify the components of drugs seized at various British music festivals was evaluated, including samples that gave inconclusive results using the conventional, on-site, spectroscopic techniques.

## 2 Materials and Methods

### 2.1 Chemicals and Samples

LC-MS grade methanol and methyl-*tert*-butyl ether (MTBE) were purchased from Rathburn (Walkerburn, U.K.). Caffeine, benzocaine, phenacetin, lidocaine hydrochloride, levamisole hydrochloride (racemic), tripelennamine hydrochloride, and quinoline solution were obtained from Sigma-Aldrich (Dorset, U.K.). Certified reference standards of MDMA, cocaine, ketamine, 2C-B, and CMC were from Chiron (Woking, U.K.). In addition, “street-drug” samples from the TICTAC collection^28^ that had been previously analyzed by benchtop GC-MS were used, e.g., paramethoxymethamphetamine (PMMA) and MDMA tablets.

Samples collected by TICTAC (*N* = 300) in the summers of 2021 and 2022 from three music festivals in the U.K. (hereafter referred to as “Festival 1”, “Festival 2”, and “Festival 3”, for confidentiality reasons) were included in the laboratory-based testing. Additionally, 94 samples were analyzed on-site at Festival 3 in the summer of 2023. Of note, these represented the samples that could not be successfully identified on-site and required to be analyzed in a laboratory environment on the benchtop GC-MS (such as new tablets, mixtures, and low-concentrated samples).

### 2.2 Instrumentation and Sample Preparation

#### 2.2.1 Laboratory-based GC–MS

Sample composition was confirmed using an Agilent 7890A GC with 5975C VL MSD (Agilent, Santa Clara, CA, U.S.A.) equipped with a split–splitless injector and an HP5-MS column (30 m × 0.25 mm, 0.25 μm film thickness). Agilent MassHunter software (version B08.00) was used for data analysis. Samples were prepared by adding 1 mL methanol to a 1.5 mL Eppendorf tube (Appleton Woods, Birmingham, U.K.) containing approximately 30 mg of powdered sample which was then vortex-mixed (10 min) and centrifuged (6030*g*, 1 min). A portion (10 μL) was then diluted with 1 mL MTBE containing internal standards (100 mg/L each quinoline and tripelennamine). Sample preparation for tablets suspected to contain 2C-B or benzodiazepine analogues was modified to include a 10-fold dilution, rather than the 100-fold dilution used for other compounds (i.e., 100 μL of extract in 1 mL of MTBE). A 1 μL aliquot was injected using a 5:1 split ratio. The GC column was held at 80 °C for 4 min following injection, and then ramped at 40 °C/min to 290 °C and held for a total analysis time of 19.25 min. As well as the lower dilution step, for samples suspected to contain benzodiazepine compounds, a different GC temperature ramp was used: the column was held at 100 °C for 4 min, ramped up at a heating rate of 40 °C/min to 310 °C for 30 min, and held to a total run time of 39.25 min. Electron ionization (EI, 70 eV) was used, and MS data were acquired over the range 40–400 *m*/*z*. All spectra were compared to Cayman, National Institute of Standards and Technology (NIST), Scientific Working Group for the Analysis of Seized Drugs (SWGDRUG), and TICTAC libraries for compound identification. Library-matching and scoring was based on EI spectral matching and GC retention times (RT).

#### 2.2.2 Radian ASAP

A Radian ASAP-MS instrument (Waters, Wilmslow, U.K.) was used, coupled with either an NM32LA (laboratory-based) or an NG Sirio (on-site) nitrogen generator (Peak Scientific, Inchinnan, U.K. and LNI Swissgas, Versoix, CH, respectively). The MS was controlled by MassLynx software (Version. 4.2), substance identification was via LiveID software (Version 2.0), and library-matching was against the Pandora library (Version 4.0, currently research use only). The sample preparation required a small amount of powder or crushed tablet (approximately 1 mg) to be dissolved in 1 mL methanol. A small volume of the resulting solution was then introduced to the MS source region on the tip of a glass capillary. Prior to analysis, each new glass capillary undertook a “bake-out” by heating without any analyte in the ionization source (600 °C, 1 min) to remove chemicals from manufacture. Once the samples were loaded onto the baked-out capillaries, they were volatilized by a stream of heated nitrogen, and the gaseous analyte molecules were then ionized by corona discharge (20 s). The total run cycle (including the bakeout, sample loading and acquisition) was approximatively 3 min. For the current project, the sample acquisition used a four-function full scan MS experiment (using the quadrupole mass analyzer) with increasing cone voltage (CV) settings to provide increased fragmentation. CVs were set to 15, 25, 35, and 50 V. Substance identification occurred either in real-time, as the spectra were produced, or in “offline matching” mode after the sample data had been acquired. In the latter case, it was possible to interrogate the MS data.

Analytes could be added to the library through open-source MSP Librarian software (Version 1.2.1). To add a new substance to the library, the sample was initially analyzed to generate a chronographic profile of the four-function experiment described above. A “clean” spectrum at each of the four CV settings was obtained and then added to the library using the MSP Librarian software to format the information correctly for the searching algorithm to use.

The match score for unknown samples was calculated from scores from the four different CV experiments. The match score was based on the LiveID algorithm using data from the four spectra and how well they each matched those in the library. The first function (lowest CV 15 V) has a default increased weighting due the required presence of the molecular ion within this function. However, this is a user definable parameter found within the LiveID software.

#### 2.2.3 Library Matching and Analyte Identification Criteria

The final match score was based on the similarity of the four CV spectra acquired for the samples compared to those present in the library. CV-weighting was defined in the user parameters, and the final number displayed as a score out of a maximum of 1000. Other parameters included “low peak filter” (%) which rejects all spectral peaks below the percentage defined relative to the base peak (largest peak) in the spectrum. The effect of this parameter is that low concentration analytes (relative to the major component) can be filtered out and not detected. However, the filter helps prevent the identification of false positives due to the removal of low intensity noise. This was set at the default value (5% of the base peak intensity). The “Library peaks of interest” parameter defines the number of ions (starting with the most intense) from the sample and library spectra used for comparison. This was also set at the default value of 5 ions per spectrum. The “Final Score Cutoff” is another user definable parameter which defines the level above which a result will be shown. The default is 800, so any match above 800 will be shown. For the off-site testing, only the results with final match scores greater than or equal to 950 were considered a positive identification. The on-site assessment was based on a final match score greater than or equal to 900, the different approach being due to an attempt to observe trace components and further investigate the false positive results.

### 2.3 Method Validation

The method validation process followed the guidelines published by the SWGDRUG and United Nations Office on Drugs and Crime (UNODC)^29,30^ and evaluated five drugs seen at the 2021 summer British festivals [MDMA, cocaine, ketamine, 2C-B, and CMC (including 3-CMC and 4-CMC)]. Validation parameters included: selectivity/specificity, limit of identification (LOI), inter- and intraday precision, carry-over, and robustness/ruggedness.

Selectivity and specificity were evaluated using (i) solutions of a range of known “cutting” agents and (ii) “street samples” of tablets found to contain structurally related substances when analyzed by GC-MS. Approximately 0.5 mg of each of the cutting agents (caffeine, benzocaine, phenacetin, lidocaine, and levamisole) were dissolved in 1 mL methanol and analyzed by the Radian ASAP-MS instrument. Street samples containing methylenedioxyethylamphetamine (MDEA), benzylpiperazine (BZP), dibenzylpiperazine (DBZP), trifluoromethylphenylpiperazine (TFMPP), paramethoxymethamphetamine (PMMA), methamphetamine, and chloroethcathinone (CEC) were crushed using an agate mortar and pestle, and approximately 0.5 mg of each substance dissolved in 1 mL methanol. These solutions/suspensions were then analyzed using the Radian ASAP-MS system. The data were compared to the GC–MS results.

For evaluating the LOI, 0.5 g/L solutions of MDMA, cocaine, ketamine, and CMC, and a 1.0 g/L solution of 2C-B (all prepared in methanol) were analyzed in triplicate. These solutions were serially diluted (1 + 1, *v/v*) and each dilution analyzed in triplicate until the average match score from the three analyses fell below 950.

Precision was evaluated using the same starting solutions used to assess the LOI. These solutions were analyzed in triplicate across three consecutive days, in the morning and afternoon, and the results assessed for agreement.

To test carryover, the starting solutions used to assess the LOI were analyzed followed by a “blank” (new capillary) run. If no residue was detected from the previous sample, then carryover was considered absent.

A total of 12 different samples were analyzed in a different laboratory previously and then reanalyzed again on the Radian ASAP in the laboratory to test the ruggedness/robustness of the method and of the instrument.

Table 1 shows a summary of all the substances that were used for each validation experiment.

**Table 1:** Summary of Compounds Used in the Validation Process for Each Individual Step

selectivity/specificity	LOI	inter-/intraday precision	carry-over	ruggedness/robustness
caffeine	MDMA	MDMA	MDMA	levamisole
benzocaine	cocaine	cocaine	cocaine	benzocaine
phenacetin	ketamine	ketamine	ketamine	lidocaine
lidocaine	CMC	CMC	CMC	caffeine
levamisole	2C-B	2C-B	2C-B	phenacetin
MDEA				caffeine, phenacetin
PMMA				cocaine
methamphetamine				5F-ADB
BZP				4-CEC
DBZP				4-CMC
TFMPP				MDMA
CEC				cyclopropyl fentanyl

## 3 Results

### 3.1 Method Validation

For selectivity/specificity, the results from the Radian ASAP for eight of the ten samples agreed fully with the GC-MS results. A tablet containing both MDEA and MDMA (as identified by GC-MS) was analyzed. The Radian ASAP also detected methylenedioxyamphetamine (MDA) in this sample. Moreover, none of the piperazine analogues were detected, with results showing a false positive for caffeine (Table 2).

**Table 2:** Results from Nine Sample Used for Selectivity/Specificity Testing with Their Respective Match Scores in Brackets

substance	test
caffeine	caffeine (992)
benzocaine	benzocaine (980)
phenacetin	phenacetin (975)
lidocaine	lidocaine (975)
levamisole	levamisole (983)
MDEA, MDMA	MDMA (997), MDA (986), MDEA (904)
BZP, DBZP, TFMPP	caffeine (996)
PMMA	PMMA (989)
methamphetamine	methamphetamine (994)

Table 3 shows the minimum drug concentrations that produced a positive identification (match score ≥950) on the portable instrument during the validation experiment. Precision studies showed consistent results across three consecutive days in the morning and afternoon with only minor variations in the match scores (Table 3). Among the five substances tested, cocaine showed the greatest variation in match score, but still did not affect overall identifications.

**Table 3:** Lowest Detected Concentrations That Rendered a Positive Identification for the Five Substances Included in the Validation Study and the Inter- and Intra-Day Precision Test Showing the Variation for Each of the Five Substances Tested

drug	LOI concentration (mg/mL)	inter- and intraday precision (%)
MDMA	0.03	1.33
cocaine	0.02	5.67
2C-B	0.03	2.67
ketamine	0.01	2.33
CMC	0.06	2.33

The carryover testing revealed that using a new, baked-out glass capillary after running a sample, regardless of its concentration, did not produce any matches to the previously tested samples. No carryover was observed in the field. However, carryover can occur during major contamination. Blank samples can be completed to determine if this is an issue, and the source can be removed and cleaned. The main area of contamination is usually the corona pin, which can be cleaned with methanol and tissue. The ruggedness/robustness assessment showed consistent results on the Radian ASAP despite different environmental conditions.

### 3.2 “Street Sample” Analysis

**Figure 1:**
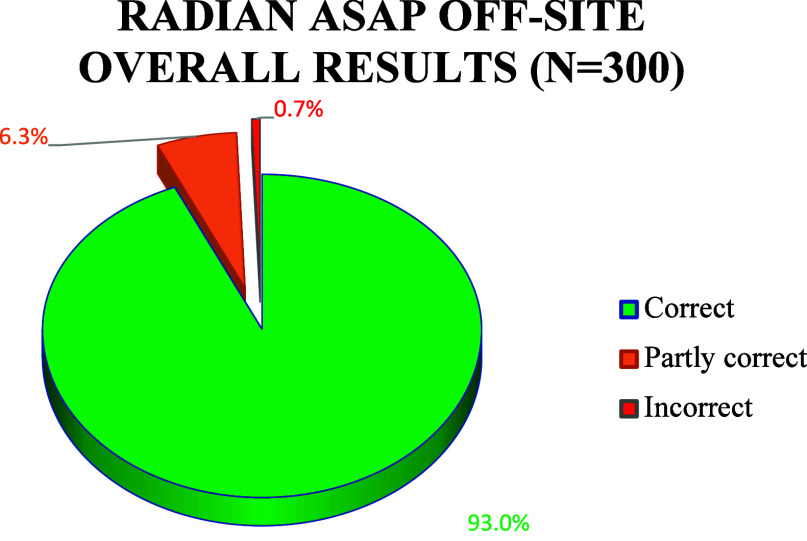
Correct, partly correct and incorrect results acquired on the Radian ASAP during the laboratory-based assessment.

A total of 300 samples originating from three different summer festivals (two occurring in 2021 and one in 2022) were analyzed in the TICTAC laboratory on the Radian ASAP unit and confirmed on the benchtop GC-MS. These included tablets that were not present in the TICTAC collection alongside powders and crystals that showed inconclusive results after attenuated total reflectance-Fourier transform infrared (ATR-FTIR) and Raman analysis. Relative to the GC-MS, the Radian ASAP produced 93% (*N* = 279) “correct” results (i.e., complete agreement with the GC-MS results), 6.3% (*N* = 19) “partly correct” results (i.e., some agreement in identification of compounds between the methods), and 0.7% (*N* = 2) “incorrect” identifications (i.e., different, or missed identifications between the methods) (Figure 1).

Of the partly correct results (Table 4), all except three samples were tablets. In eight tablets that contained MDMA and 2C-B according to the GC-MS results, the Radian ASAP only detected and correctly identified 2C-B (Table 4, Samples 12–19 inclusive). For these eight tablets, the mean (range) peak area ratio of MDMA to 2C-B by GC-MS was 0.7 (0.1–1.2), suggesting low amounts of MDMA when compared to the typical dose of a tablet that only contains MDMA, but high enough concentration that should have been detected. Similarly, it was not able to detect MDMA or caffeine in three tablets that contained MDMA, caffeine and 2C-B by GC-MS. Furthermore, the Radian ASAP unit also failed to detect the caffeine in a tablet that contained MDMA and caffeine by GC-MS. These analyses also missed some other cutting agents such as paracetamol (*N* = 1) and benzocaine (*N* = 2). However, in a different sample, that contained *N*-ethylpentylone and caffeine by GC-MS, the Radian ASAP only identified caffeine. Finally, a sample containing methamphetamine, mephedrone and caffeine by GC-MS was identified as containing caffeine and mephedrone on the Radian ASAP. The two incorrect results showed no active ingredients on the Radian ASAP, although they gave positive identifications for psilocin and bromazolam, respectively, on the benchtop GC-MS (Table 4) - see the Discussion section for the explanation of these observations. A clear representation of the specificity and sensitivity of the method at various match score values, evaluating the true positive (TP), false positive (FP), true negative (TN), and false negative (FN) results compared to the GC-MS is shown in Table S1. A match score of 925 and below displays a high rate of false positive results, while at 975 or above a high rate of FN results is recorded (i.e., missing drugs). No FP and no FN results are shown at a match score of 950.

**Table 4:** Summary of “Partly Correct” (*N* = 19, Samples 1–19) and “Incorrect” (*N* = 2, Samples 20–21) Results Acquired in the TICTAC Laboratory Relative to Those Identified by GC-MS

sample no.	sample form	GC-MS results	Radian ASAP results (mass match score)	comments	final result
1	powder	paracetamol, ketamine, cocaine	**cocaine** (995), **ketamine** (988)	false negative: paracetamol	partly correct
2	tablet	caffeine, *N*-ethylpentylone	**caffeine** (986)	false negative: *N*-ethylpentylone, NB: this was in the library	partly correct
3	tablet	MDMA, caffeine	**MDMA** (988), MDA (947)	false negative: caffeine	partly correct
4	powder	benzocaine, phenacetin, cocaine	**cocaine** (995), **phenacetin** (955), benzocaine (892)	false negative: benzocaine, NB: detected but the match score was below 950	partly correct
5	powder	benzocaine, phenacetin, cocaine	**cocaine** (995), **phenacetin** (954)	false negative: benzocaine	partly correct
6	tablet	eutylone, caffeine	**eutylone** (996), pentylone (938)	false negative: caffeine	partly correct
7	tablet	MDMA, caffeine, ketamine	**ketamine** (997), **MDMA** (955), MDA (909)	false negative: caffeine	partly correct
8	tablet	methamphetamine, mephedrone, caffeine	**caffeine** (994), **mephedrone** (988)	false negative: methamphetamine	partly correct
9	tablet	MDMA, 2C-B, caffeine	**2C-B** (998)	false negative: MDMA and caffeine, NB: additional concentration step for 2C-B by GC-MS only	partly correct
10	tablet	MDMA, 2C-B, caffeine	**2C-B** (996)	false negative: MDMA and caffeine, NB: additional concentration step for 2C-B by GC-MS only	partly correct
11	tablet	MDMA, 2C-B, caffeine	**2C-B** (996)	false negative: MDMA and caffeine, NB: additional concentration step for 2C-B by GC-MS only	partly correct
12	tablet	MDMA, 2C-B	**2C-B** (998)	false negative: MDMA, NB: additional concentration step for 2C-B by GC-MS only	partly correct
13	tablet	MDMA, 2C-B	**2C-B** (998)	false negative: MDMA, NB: additional concentration step for 2C-B by GC-MS only	partly correct
14	tablet	MDMA, 2C-B	**2C-B** (989)	false negative: MDMA, NB: additional concentration step for 2C-B by GC-MS only	partly correct
15	tablet	MDMA, 2C-B	**2C-B** (998)	false negative: MDMA, NB: additional concentration step for 2C-B by GC-MS only	partly correct
16	tablet	MDMA, 2C-B	**2C-B** (993)	false negative: MDMA, NB: additional concentration step for 2C-B by GC-MS only	partly correct
17	tablet	MDMA, 2C-B	**2C-B** (995)	false negative: MDMA, NB: additional concentration step for 2C-B by GC-MS only	partly correct
18	tablet	MDMA, 2C-B	**2C-B** (999)	false negative: MDMA, NB: additional concentration step for 2C-B by GC-MS only	partly correct
19	tablet	MDMA, 2C-B	**2C-B** (988)	false negative: MDMA, NB: additional concentration step for 2C-B by GC-MS only	partly correct
20	capsule	psilocin	NDD	false negative: psilocin, NB: this was in the library	incorrect
21	tablet	bromazolam	NDD	false negative: bromazolam, NB: this was not in the library	incorrect

The results acquired on the Radian ASAP in the field (*N* = 94) at a music festival in the summer of 2023, 92.5% were consistent with the GC-MS results.

**Figure 2:**
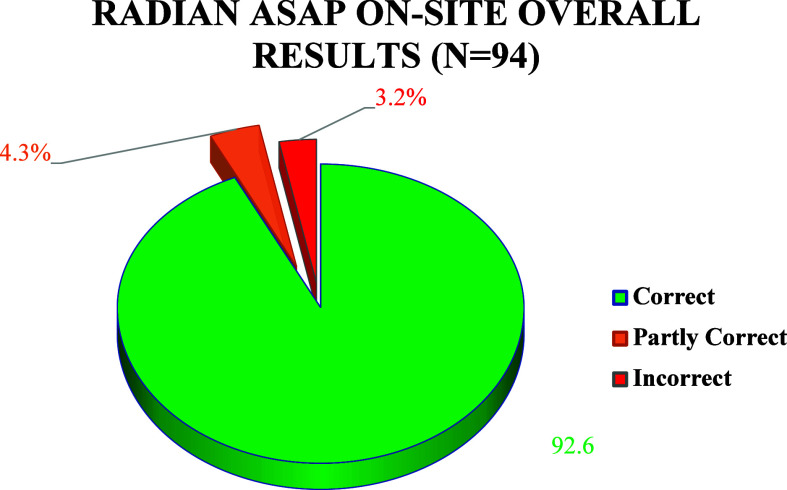
Correct, partly correct, and incorrect results acquired on the Radian ASAP instrument on-site at Festival 3 in the summer of 2023.

As shown in Figure 2, a proportion of 4.3% (*N* = 4) fell under the “partly correct” results, the Radian identifying an extra active ingredient in two samples and missing one or two components in three samples (Table 5). The incorrect results (*N* = 3) consisted of two false negatives: 4-acetoxy-methylethyl-tryptamine (4-AcO-MET) and 4-hydroxy-methylethyl-tryptamine (4-OH-MET)—not present in the library; and a false positive: delta-9-tetrahydrocannabinol (THC) as shown in Table 5.

**Table 5:** Summary of “Partly Correct” (*N* = 4, Samples 1-4) and “Incorrect” (*N* = 3, Samples 5–7) Results Acquired on-Site Relative to Those Identified by GC-MS

sample no.	sample form	GC-MS results	Radian-ASAP results	comments	final result
1	crystals	ketamine, benzocaine, dimethylsufone	**ketamine** (989), **lidocaine** (982)	false negative: benzocaine and dimethylsulfone, false positive: lidocaine	partly correct
2	herbal	nicotine, THC	**THC** (989)	false negative: nicotine, NB: nicotine not in the library	partly correct
3	powder	cocaine, benzocaine	**cocaine** (999), **levamisole** (963), benzocaine (932)	false positive: levamisole	partly correct
4	powder	MDMA, ketamine, traces of 2C-B	**ketamine** (996), MDMA (928)	false negative: 2C-B and MDMA below the threshold	partly correct
5	herbal	NDD	**THC** (973)	false positive: THC, but the next two runs gave no response for THC, therefore it was contamination from the previous sample	incorrect
6	powder	4-AcO-MET	NDD	false negative: 4-AcO-MET, NB: not in the library	incorrect
7	powder	4-HO-MET	NDD	false negative: 4-HO-MET, NB: not in the library	incorrect

## 4 Discussion

### 4.1 Method Validation

**Figure 3:**
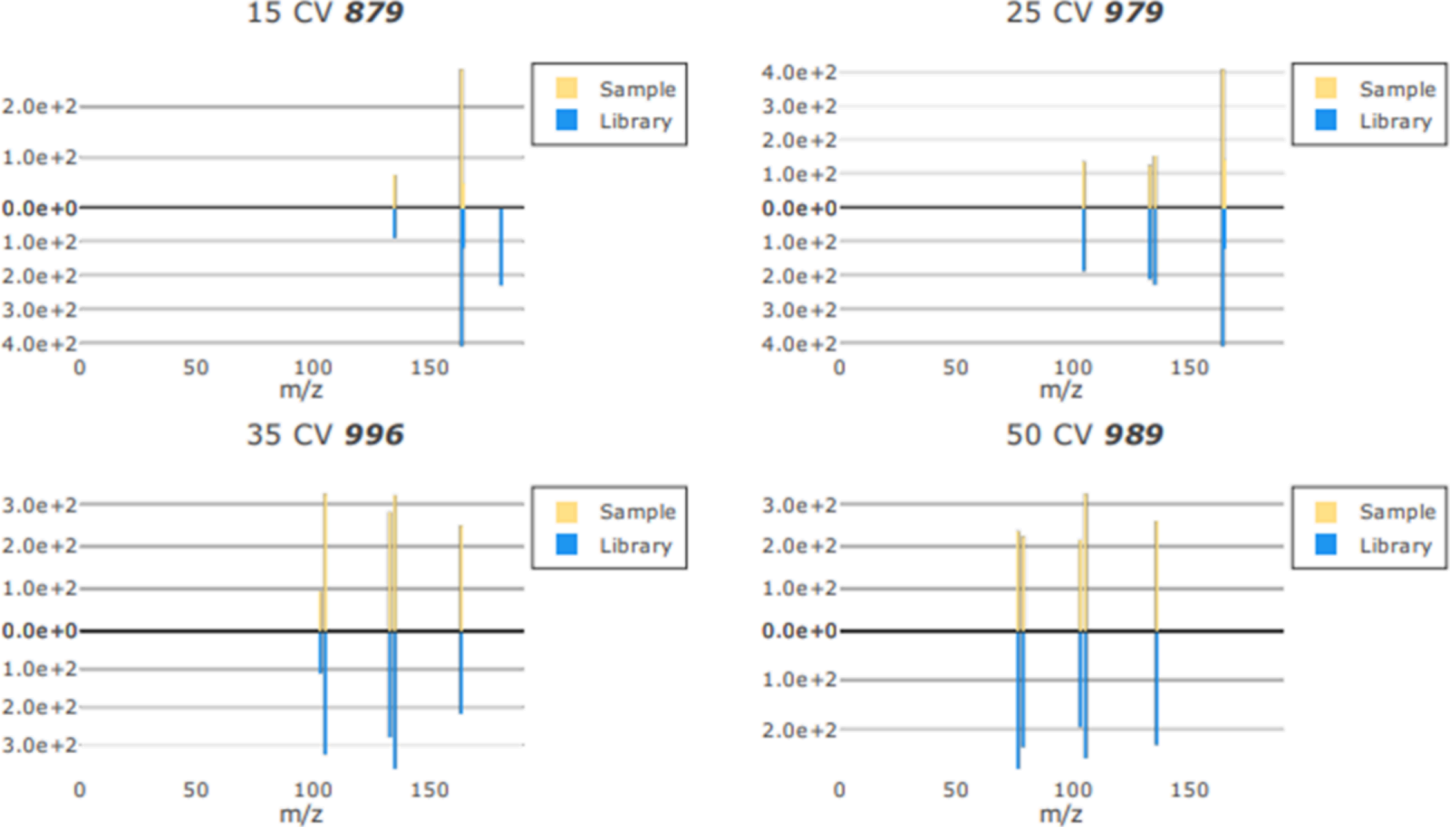
Example of MDA ions for 15, 25, 35, and 50 CVs with comparisons to library ions.

The only validation step that seemed to challenge the use of the Radian ASAP for illicit drug analysis was the selectivity/specificity test in the presence of structurally related compounds. The Radian unit failed to identify MDEA (match score 904) and detected MDA instead. The reason for the latter could be due to the close structural similarity between MDMA and MDA, while the low match score for MDEA could be explained by the smaller amount of MDEA in that specific sample (MDMA:MDEA ratio = 3.7). The values for the 25, 35, and 50 CV scores for MDA were consistently over the 950 threshold (Figure 3).

However, the 15 CV which carries the most weighting when calculating the match score was significantly lower, leading to the overall match scores being <950 due to missing the molecular ion (*m*/*z* 180). This may be in relation to the additional methyl group in MDMA which is not present in MDA which would be best represented in the 15 CV condition where the least fragmentation occurs. This may not be important as MDA and MDMA have similar interactions with neurotransmitters and have been assumed to be virtually identical in action.^31^ According to Bade et al., they have been found to be the most popular drugs at festivals. However, because MDA is a metabolite of MDMA the abundance of MDA has been difficult to quantify from wastewater analysis. Therefore, the two drugs are frequently discussed together in studies due to the inability to differentiate them as the source or metabolite.^32^

However, the false positive result for closely related compounds was an issue for other pairs of compounds, such as amphetamine and methamphetamine and eutylone and pentylone. Although not considered a major predicament from a harm reduction point of view, this becomes a problem in the forensic field and the software possibly needs to be improved to attempt to remove these false positive results.

The specificity/selectivity testing showed that the Radian ASAP could not discriminate any of the three piperazines that were present in a single tablet as identified by GC-MS. Although it can be classified as a sensitivity drawback, this is the opposite of what Fabregat-Safont et al. concluded using the same instrument.^33^ Their study showed that a few novel psychoactive substances (NPS), such as cathinones and synthetic cannabinoids, could be identified even in trace amounts, on fingers, after just touching the substances and washing the hands before sampling. However, as Boronat Ena et al. suggested, the ASAP technique has not been extensively used to analyze drugs of abuse,^34^ hence more work is needed to identify all the possible hurdles and limitations.

### 4.2 Testing Batch Results

Although the Radian ASAP has been briefly assessed by another research group and proved to be successful in the identification of MDMA, ketamine, cocaine, 2C-B, flualprazolam, paracetamol, and caffeine,^35^ it has not been fully validated and its on-site analysis ability has not been evaluated previously. However, its potential for quantification of melatonin in over-the-counter hypnotics was shown to be a rapid, reliable, and cost-effective alternative to conventional methods.

Having achieved 93% and 92.5% correct results off and on-site, respectively, it can be concluded that the Radian ASAP is a useful additional technique that can be deployed for the rapid, in-field analysis of recreationally abused drugs. As supported by Mistry et al.,^35^ this method can assist in analyzing tablets, mixtures, and low-concentration samples, which may produce inconclusive results with ATR-FTIR and Raman instruments. It is of note that none of the correct, partly correct, and even incorrect results presented in this study were successfully confirmed using any of the spectroscopic methods employed for on-site drug analysis by TICTAC. However, the partly correct and incorrect results make the Radian ASAP a less viable tool for forensic purposes where confirmatory analysis with a chromatographic step would still be required.

**Figure 4:**
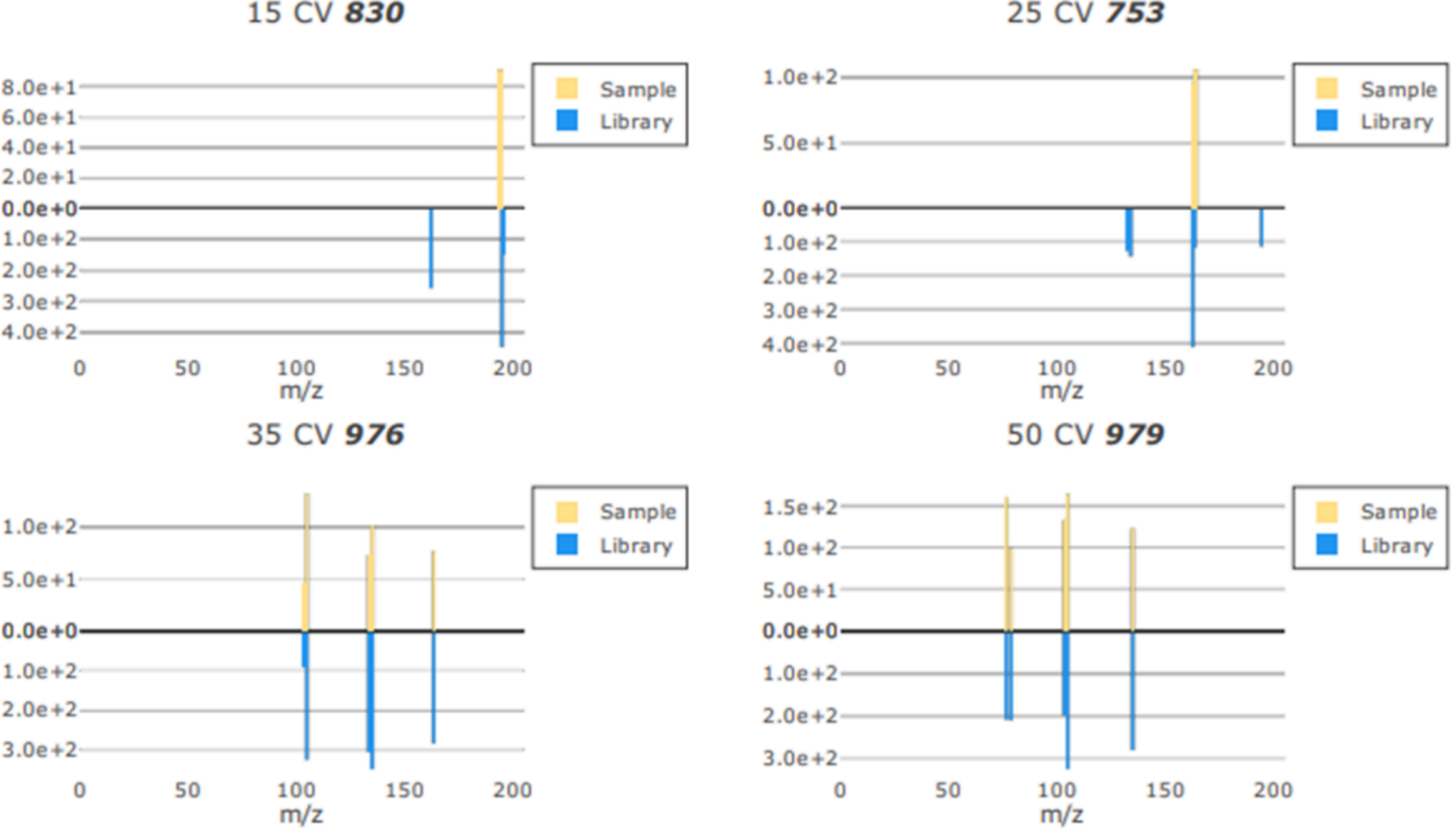
Example of MDMA ions for 15, 25, 35, and 50 CVs with comparisons to library ions for a match score <950 (874) in a 2C-B sample that contained MDMA as well.

Eight tablets reported as containing 2C-B only were found to contain also MDMA according to GC-MS analysis; this could be due to a difference in sensitivity between the two instruments but also due to the different sample preparation dilution factor on the GC-MS only. Examination of the ion matches produced by LiveID that form the basis of the CV scores shows that for the 2C-B samples that also contained MDMA there was a missing ion in the 15 CV spectra for MDMA (Figure 4).

This may be because of the low relative abundance or, as the missing ion does not display a high intensity, a weak intensity may have been removed by the 5% low peak filter leading to a match score <950 for MDMA. Although the amount of MDMA has not been determined, the GC-MS peak (and its peak area) suggested MDMA was present at very low concentration compared to its typical dose in MDMA only tablets. Similarly, three tablets that contained 2C-B, and low amounts of MDMA and caffeine by GC-MS gave a positive for 2C-B only on the Radian ASAP. While the aspect of the tablet is specific to 2C-B only, it is possible that the contamination occurred during the manufacturing process. As highlighted by Scott and Scott, there is no certainty that one sample is representative of the whole batch as manufacturing illicit substances lacks quality control. Moreover, they emphasize that the content can vary and be different than expected or sold as, therefore MDMA being present in 2C-B tablets may come as no surprise from a research standpoint, but it could pose potential risks for users.^8^

The Radian ASAP failed to identify a few other cutting agents, such as caffeine, benzocaine and paracetamol (partly correct results), although these were detected by the GC-MS. It can be argued that, from a harm reduction perspective, the presence of cutting agents may not be crucial as the active ingredient is the one that shapes prevention advice. However, as emphasized by Fioretin et al., the undesired effects can be dependent on the nature, the number, and the amount of the cutting agents.^19^ For instance, high doses of paracetamol can lead to liver damage and many other adverse effects especially in poly drug use, while caffeine could trigger headaches, irritability, anxiety, mood, and sleep disturbances. Therefore, the ability to detect all substances present within a sample remains relevant for the information and prevention services. The data corroborated by Valente et al. in 2016 at Boom Festival in Portugal shows that people adapt their drug use choices and patterns based on the drug checking results and advice.^36^ Therefore, there is a clear need for accurate and fast analytical results to design better prevention and harm reduction programmes. Missing the *N*-ethylpentylone in one of the samples and only detecting the cutting agent (caffeine) could lead to more serious complications. According to Pascoe et al., MDMA was detected in 92.8% of samples in 2019, with an almost insignificant percentage of cathinones (<1%), while in 2021, MDMA presence fell to 54.8%, and the main component in 19.8% of samples was a synthetic cathinone.^37^ Although *N*-ethylpentylone is not as common currently, there is still a lack of knowledge regarding its pharmacology and toxicology. As stated by Ikeji et al. in their case report, *N*-ethylpentylone seems to adversely damage several organs, causing cardiac arrest, rhabdomyolysis, renal failure, hepatic failure, anoxic brain injury, coagulopathy, and death,^38^ hence its identification is paramount.

**Figure 5:**
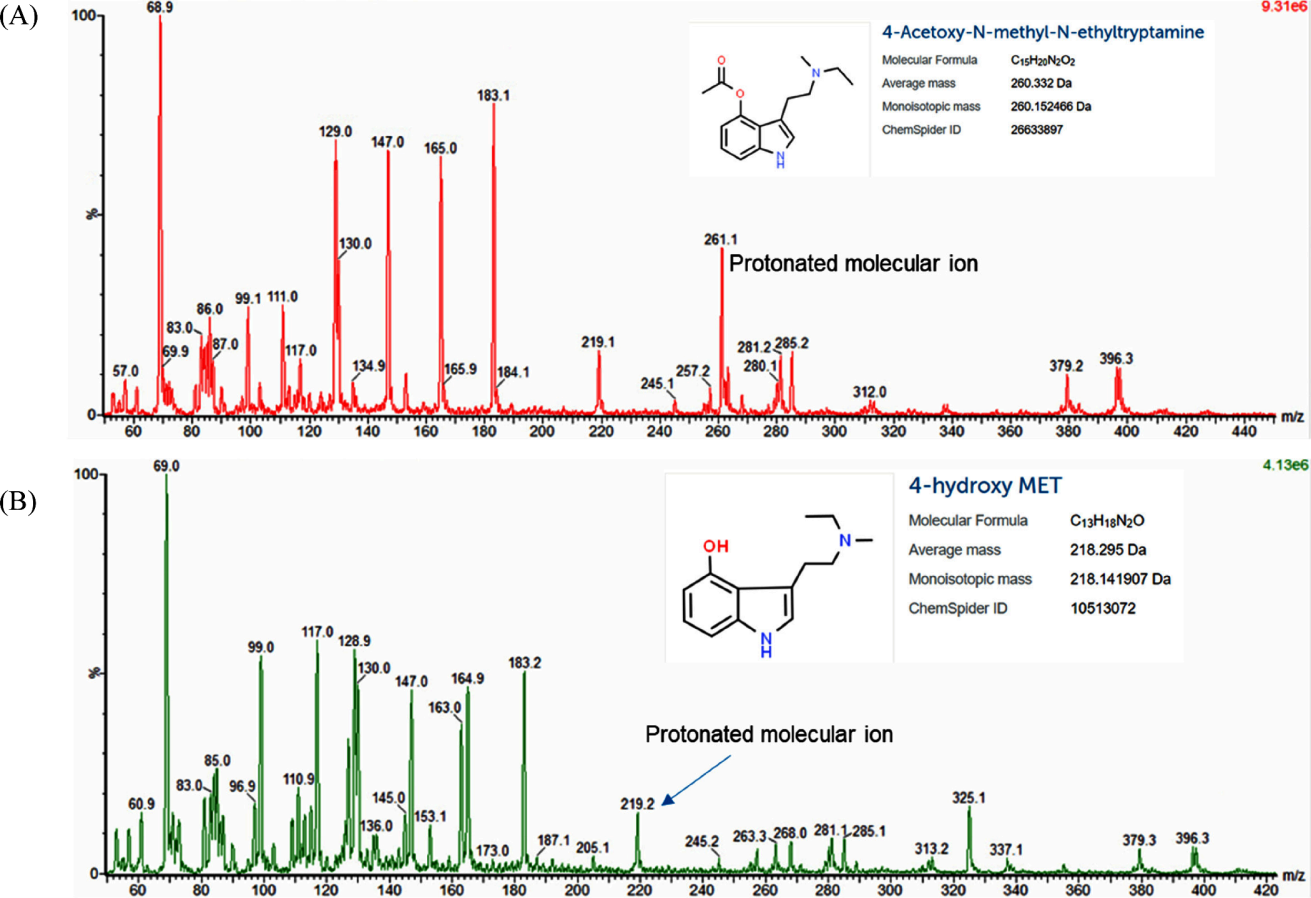
MS results acquired on the Radian ASAP for 4-AcO-MET (A) and 4-HO-MET (B) that show the presence of the two tryptamines in the samples.

With regard to the incorrect results obtained off- and on-site (0.7 and 3.2%, respectively), these were partly due to the library coverage. As part of the laboratory-based assessment, bromazolam was not identified because this was not included in the Pandora library. However, psilocin should have been identified as the oral dose needed to cause an altered state of consciousness is 12–20 mg.^39^ Review of the Radian data showed that at the time this sample was analyzed, the ion source environment was heavily contaminated, and the match score was therefore significantly reduced. When evaluated in the field, the Radian ASAP gave a false positive result for delta-9-THC, due to analysis of a previous “oily” vape cannabis sample that had contaminated the system. Subsequent analysis of this false positive sample produced no THC response showing the initial result being carryover from the previous high intensity “oily” sample. These two samples in particular suggest that although the use of new, baked out capillaries prevents carryover, it is possible that the ionization source itself may become contaminated following analysis of some materials, and this may need to be considered (i.e., ion source cleaning). However, the heating of the source environment to a maximum can be undertaken to remove the carryover in most circumstances. It also failed to identify two tryptamine analogues (4-AcO-MET and 4-OH-MET) which, as previously stated, were not present in the library. However, a thorough examination of the mass spectra detected the molecular ions for both hallucinogens; therefore, it is believed that this could have led to a successful identification were they to be present in the library (Figure 5). Due to time and cost restrictions, the library update for the two hallucinogens was not feasible. Therefore, this can be assigned for future work.

**Figure 6:**
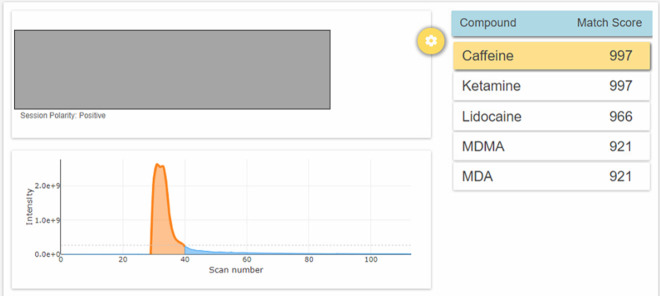
Radian ASAP results showing its ability to correctly identify five different compounds (caffeine, ketamine, lidocaine, MDMA, and MDA)—albeit the latter one is a false positive—in a mixed sample–a pink powder sold as “Tusi” or pink cocaine.

The employment of the Radian ASAP for field testing has some great advantages allowing for drug analysis of mixtures, low and very low concentration samples (such as lysergic acid diethylamide—LSD blotters and other potent substances), and tablets. It proved to be successful with very complex mixtures being able to correctly identify a “Tusi” sample when used on-site (Figure 6 and Figures S1–S5), which was of great help to medical and welfare teams. The mixture, also known as pink cocaine (referred to “cocaina rosada” in Spanish) rarely contains cocaine, as supported by the current study, being reported by other peers as a concoction of ketamine and MDMA with or without additional drugs.^40^

The Radian ASAP, while not suitable for quantitative drug analysis, has proven to be a quick and accurate way to qualitatively analyze drugs used by festival attendees. This can be a crucial tool in improving risk management.

## 5 Conclusions

Controlled drugs are known to be used in various party settings, but more predominantly at music festivals, where their abuse has led to an increased number of hospitalisations and even deaths.^3,41^ Although England’s drug and alcohol treatment system has been shaped over the years by fiscal constraints, the increase in drug-related deaths pushed the new drug strategy to commit more funds for community treatment and recovery services. According to the Department of Health and Social Care, the annual expenditure of £680 million requires additional funding to deliver high-quality drug prevention and treatment services.^42^

This study shows that the Radian ASAP instrument can be successfully used off- and on-site for drug testing analysis with approximately 93% of results in agreement with an established GC-MS method. Of the few differences that were observed between the methods, most could be easily explained. The purpose of the investigation was to assist the available and popular spectroscopic techniques and overcome limitations such as analysis of mixtures, tablets, and low concentration samples. The qualitative analytical method was successfully validated for four of the most common traditional drugs of abuse and a synthetic cathinone. The validation process identified some limitations around selectivity/specificity. The presence of false positive results for structurally similar compounds needs to be addressed in future research to render this technique viable also in the forensic field and expansion of the library will be required to detect a wider range of NPS alongside the traditional and common drugs of abuse.

Nevertheless, the Radian ASAP technology has the potential to be used for on-site identification of substances at festivals and in healthcare and police settings, where quick identification by nonexperts can help determine the most appropriate treatment or course of action.

## Supporting Information Available

Figures S1–S5 show the individual, four CVs fragmentations of caffeine, lidocaine, ketamine, MDMA, and MDA identified in a pink powder known as “Tusi” (PDF)
