# Supplementary material for: Assessment of a Single Quadrupole Mass Spectrometer Combined with an Atmospheric Solids Analysis Probe for the On-Site Identification of Amnesty Bin Drugs
Source: J Am Soc Mass Spectrom. 2024 Jun 5;35(7):1480–9. doi: 10.1021/jasms.4c00064 (PMC11228975; doi:10.1021/jasms.4c00064)
Supplement: Supplementary file 1 — js4c00064_si_001.pdf [file js4c00064_si_001.pdf]

Supporting Information

Assessment of a Single Quadrupole Mass Spectrometer Combined with an Atmospheric Solids Analysis Probe for the On-site Identification of Amnesty Bin Drugs

Anca Frinculescu[a,b], Benjamin Mercer[c], Trevor Shine[b], John Ramsey[b], Lewis Couchman[a,d], David Douce[e], Nunzianda Frascione[a], Vincenzo Abbate[a]*

[a] Department of Analytical, Environmental and Forensic Sciences, King's College London, 150 Stamford Street, London SE1 9NH, United Kingdom

[b] TICTAC Communications Ltd., Room 1.159 Jenner Wing, St. George's University of London, Cranmer Terrace, London SW17 0RE, United Kingdom

[c] Clinical Pharmacology, William Harvey Research Institute, Queen Mary University of London, London EC1M 6BQ, United Kingdom

[d] Analytical Services International, St. George's University of London, Cranmer Terrace, London SW17 0RE, United Kingdom

e Waters Corporation, Stamford Avenue, Wilmslow SK9 4AX, United Kingdom

[*] vincenzo.abbate@kcl.ac.uk

Table S1

Match Score Threshold	TP	TN	FP	FN
**800**	372	0	294	0
850	372	120	174	0
900	372	156	138	0
925	372	164	130	0
950	372	294	0	0
975	319	294	0	53

Table S1. Frequency of true positive (TP), true negative (TN), false positive (FP) and false negative (FN) results compared to GC-MS with increasing Radian ASAP match score thresholds.

Caffeine

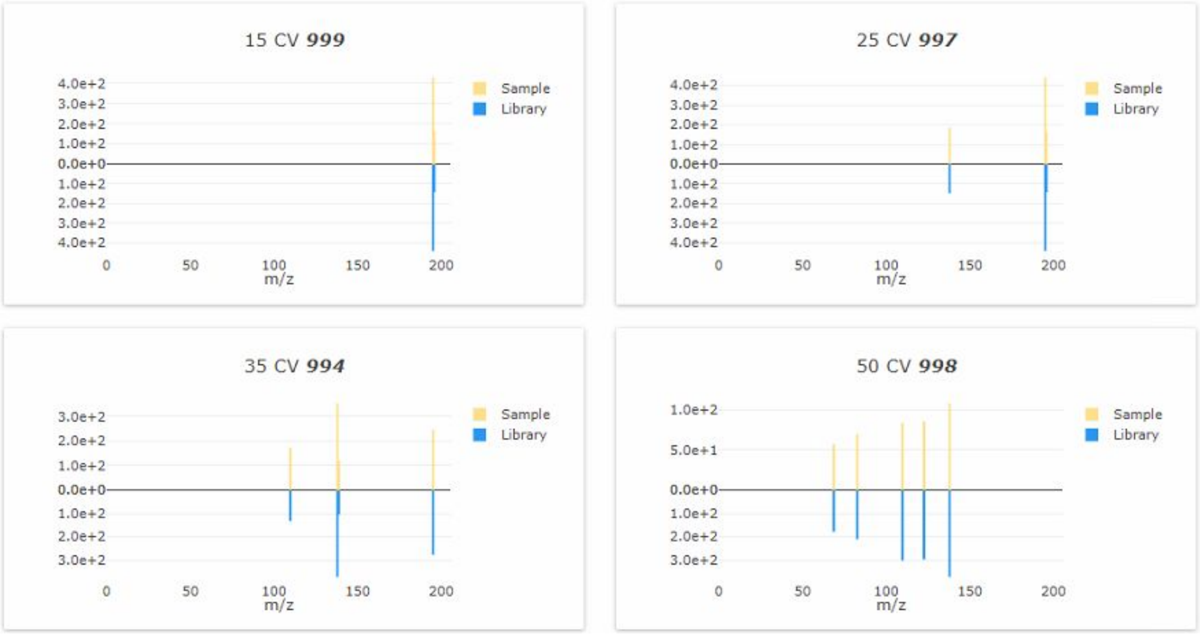

Figure S1: Caffeine fragmentation for 15, 25, 35 and 50 CVs with comparisons to library ions for a match score >900 (997) in a pink powder called 'Tusi'. The analysis was performed on-site at one of the festivals.

Ketamine

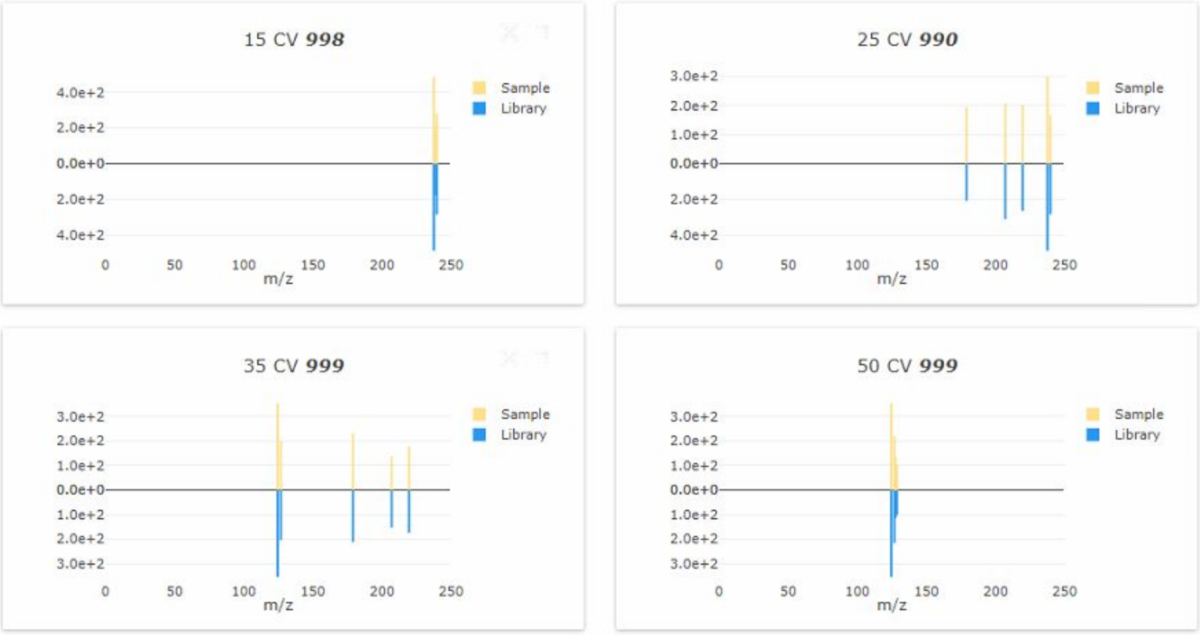

Figure S2: Ketamine fragmentation for 15, 25, 35 and 50 CVs with comparisons to library ions for a match score >900 (997) in a pink powder called 'Tusi'. The analysis was performed on-site at one of the festivals.

Lidocaine

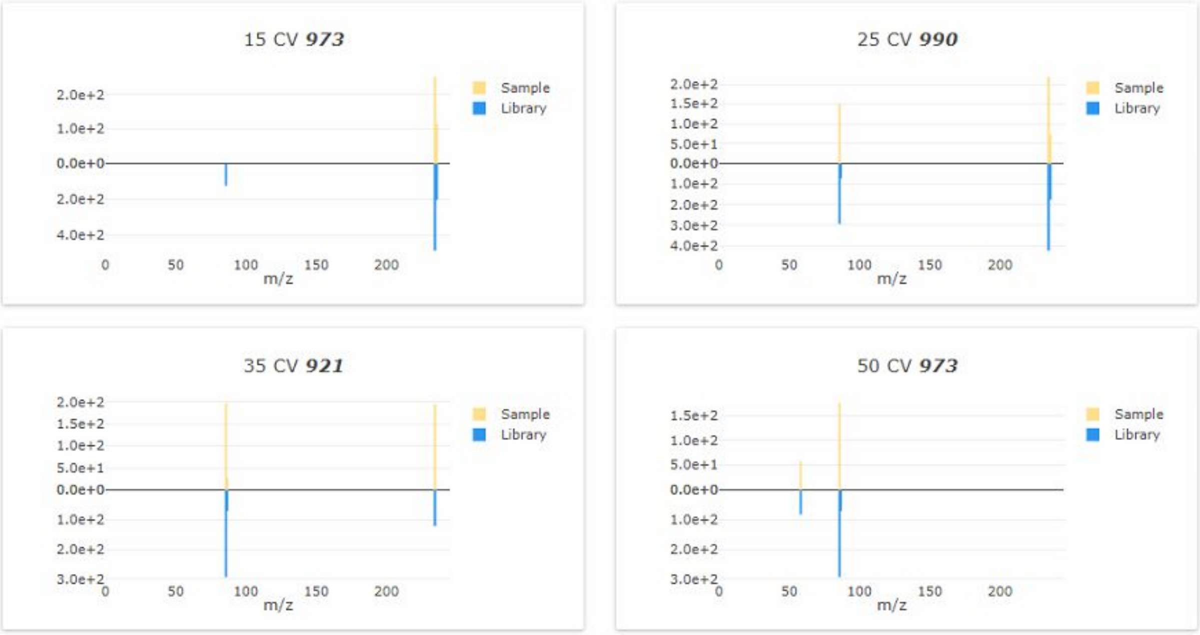

Figure S3: Lidocaine fragmentation for 15, 25, 35 and 50 CVs with comparisons to library ions for a match score >900 (966) in a pink powder called 'Tusi'. The analysis was performed on-site at one of the festivals.

MDMA

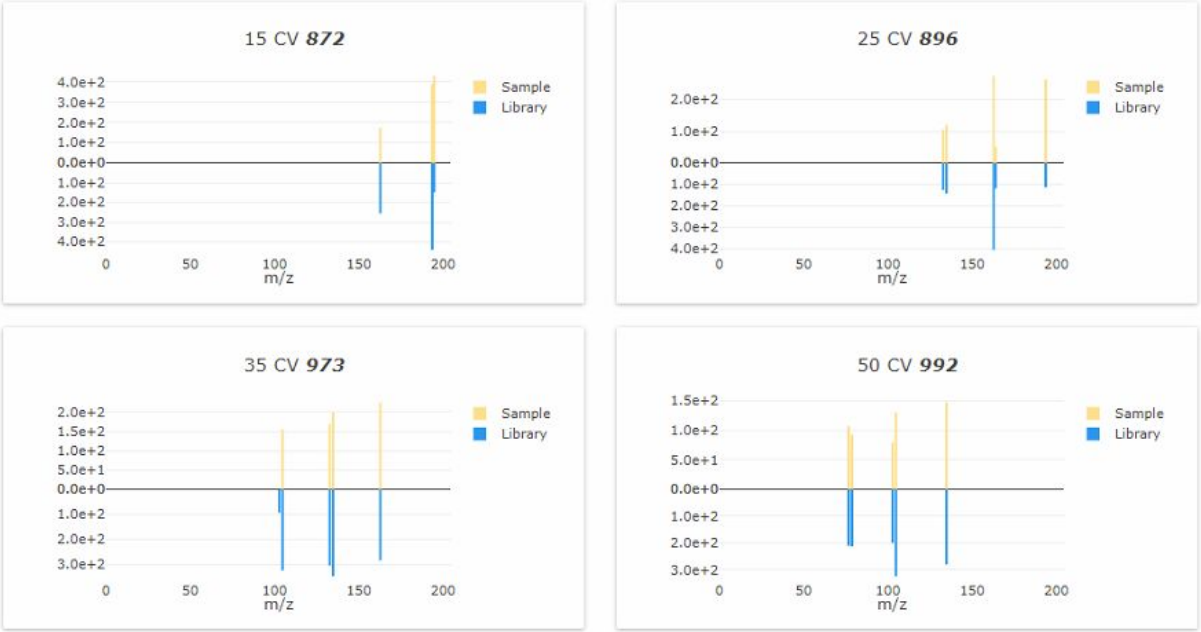

Figure S4: MDMA fragmentation for 15, 25, 35 and 50 CVs with comparisons to library ions for a match score >900 (921) in a pink powder called 'Tusi'. The analysis was performed on-site at one of the festivals.

MDA

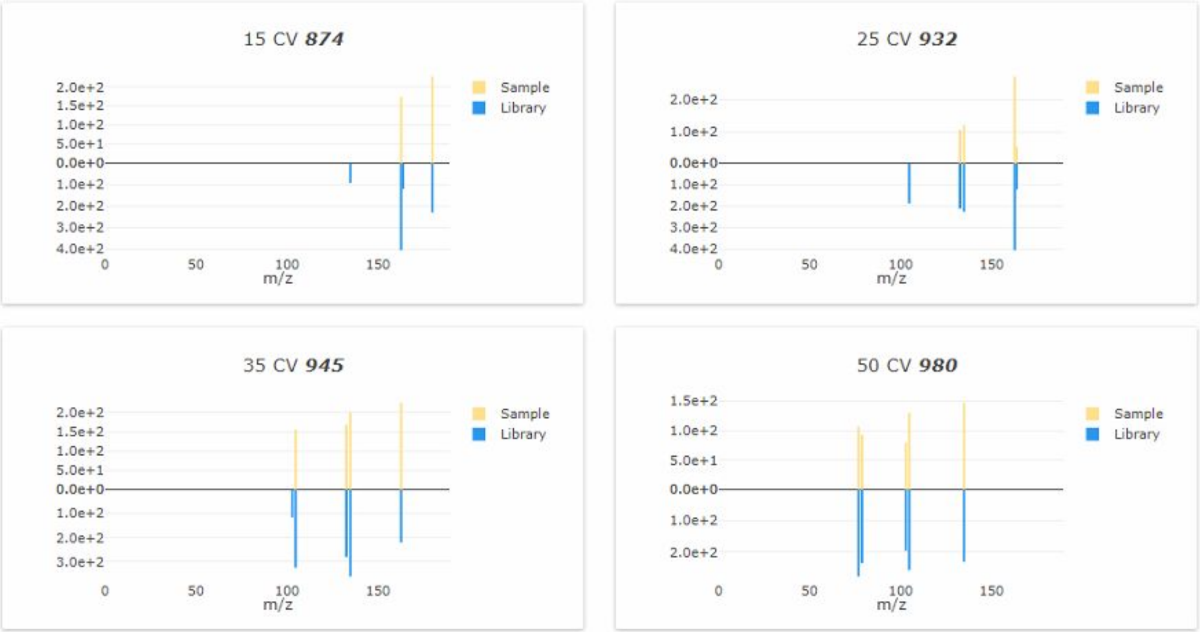

Figure S5: MDA fragmentation for 15, 25, 35 and 50 CVs with comparisons to library ions for a match score >900 (921) in a pink powder called 'Tusi' – false positive result. The analysis was performed on-site at one of the festivals.
